# Genetic characterization of *Bartonella henselae* samples isolated from stray cats by multi-locus sequence typing

**DOI:** 10.1186/s12917-023-03748-4

**Published:** 2023-10-07

**Authors:** Hüseyin Can, Mervenur Güvendi, Ecem Sürgeç, Ahmet Efe Köseoğlu, Sedef Erkunt Alak, Muhammet Karakavuk, Aytül Gül, Mert Döşkaya, Adnan Yüksel Gürüz, Cemal Ün, Aysu Değirmenci Döşkaya

**Affiliations:** 1 Faculty of Science, Department of Biology Molecular Biology Section, Ege University, İzmir, Turkey; 2 Faculty of Science, Department of Biology Zoology Section, Ege University, İzmir, Turkey; 3 Faculty of Engineering and Natural Sciences, Department of Molecular Biology and Genetics, Biruni University, İstanbul, Turkey; 4 Ege University Vaccine Development Application and Research Center, İzmir, Turkey; 5 Ege University Ödemiş Vocational School, İzmir, Turkey; 6 Faculty of Engineering, Department of Bioengineering, Ege University, İzmir, Turkey; 7 Department of Bioengineering, Ege University Graduate School of Natural and Applied Sciences, Izmir, Turkey; 8 Faculty of Medicine, Department of Parasitology, Ege University, İzmir, Turkey

**Keywords:** *B. henselae*, Genotyping, Genetic diversity, New sequence type

## Abstract

**Background:**

*Bartonella henselae* is one of the most commonly identified *Bartonella* species associated with several human diseases. Although *B. henselae* was detected in humans and cats in Turkey, they have not been genotyped previously. Therefore, this study aimed to genotype *B. henselae* samples (n = 44) isolated from stray cats using the multi-locus sequence typing (MLST) method. For this aim, eight different housekeeping markers were amplified by nested PCR and then sequenced to reveal sequence types (STs) of *B. henselae* samples.

**Results:**

Allelic profiles obtained from 40 *B. henselae* isolates (90.9%) were compatible with available allelic profiles in the MLST online database. However, allelic profiles obtained from the remaining 4 *B. henselae* isolates (9.1%) were incompatible with the database. Among *B. henselae* isolates with compatible allelic profiles, 5 different STs including ST1, ST5, ST9, ST35 and ST36 were identified according to the *B. henselae* MLST online database. ST35 was the most prevalent ST with a prevalence rate of 29.5% (13/44), followed by ST36 with a prevalence rate of 22.7% (10/44). In addition, ST5 (16%, 7/44) and ST9 (18.2%, 8/44) were also among the prevalent STs. The prevalence of ST1 was 4.5% (2/44). For *B. henselae* isolates with incompatible allelic profiles, we recommended a new ST called ST38.

**Conclusion:**

The present study genotyped *B. henselae* samples isolated from stray cats in Turkey for the first time and ST1, ST5, ST9, ST35, and ST36 as well as a new sequence type named ST38 were identified among these *B. henselae* isolates.

**Supplementary Information:**

The online version contains supplementary material available at 10.1186/s12917-023-03748-4.

## Background

*Bartonella* spp. are vector-borne pathogens that successfully infect many mammals including humans. Of the 38 defined *Bartonella* species, at least 18 have been stated to be zoonotic [1]. The most common species associated with human diseases are *Bartonella henselae* (*B. henselae*), *B. clarridgeiae*, *B. quintana*, and *B. bacilliformis* [2, 3]. Cat-scratch disease is caused by *B. henselae* and *B. clarridgeiae*, whereas trench fever disease is caused by *B. quintana*. Both diseases are known as bartonellosis, and symptoms include fever, bacteremia, bacillary angiomatosis, and endocarditis. Sporadic cases of endocarditis in humans have also been associated with *B. koehlerae*, *B. elizabethae*, and *B. alsatica* [3–5].

The main reservoir is the domestic cat for *B. henselae*, *B. clarridgeiae*, and *B. koehlerae* [5] while cat fleas (*Ctenocephalides felis*) is their natural vector. Additionally, *B. rochalimae*, *B. elizabethae*, *B. quintana* and *B. grahamii* have been found in cats [6]. Although *Bartonella* species can infect cats, no symptoms generally occur. However, *B. henselae* infection has been linked to uveitis and endocarditis in cats, and some symptoms including lymphadenopathy, fever, and neurological signs have been reported in experimentally infected cats [7, 8]. To date, different seroprevalence rates varying from 0 to 80% for *Bartonella* spp. in cats have been reported [9, 10]. Our previous study also detected a prevalence rate of 12.5% in stray cats for *Bartonella* spp. by a nested PCR targeting the 16-23 S internal transcribed spacer gene (ITS) [11].

To reveal the genetic diversity of *B. henselae* samples detected in humans or cats, genotyping studies have been performed by multi-locus sequence typing (MLTS) where *16 S rRNA*, *batR*, *ftsZ*, *gltA*, *groEL*, *nlpD*, *ribC*, and *rpoB* genes are analyzed. Until now, 37 sequence types (STs) have been identified [1] and among these STs, some of them such as ST1, ST2, ST5 and ST8 have been associated with human diseases while others including ST6 and ST7 were mainly detected in cats. In addition, ST5 and S9 have also been associated with feline infection in Spain [12].

Although several studies investigating the presence of *Bartonella* spp. in cats have been conducted in Turkey, genotype profiles of *B. henselae* isolates are not revealed. Therefore, this study aimed to genotype *B. henselae* isolates previously detected in stray cats living in İzmir, Turkey [11] using MLST analysis.

## Results

Multi-locus sequence typing based on *16 S rRNA*, *batR*, *ftsZ*, *gltA*, *groEL*, *nlpD*, *ribC*, and *rpoB* genes was achieved in 44 *B. henselae* samples isolated from stray cats. All genes were successfully amplified from 44 *B. henselae* isolates and then sequenced. According to the obtained results, *16 S rRNA* (allele 1 and 2), *batR* (allele 1 and 7) and *groEL* (allele 1 and 2) genes were represented with two different alleles while *ftsZ* (allele 1), *gltA* (allele 1), *nlpD* (allele 1), *ribC* (allele 1), and *rpoB* (allele 1) genes were represented with a single allele (Table 1).

Allelic profiles obtained from 40 *B. henselae* isolates (90.9%) were compatible with available allelic profiles in the database and STs of these samples were successfully identified. However, allelic profiles obtained from the remaining 4 *B. henselae* isolates (9.1%) were incompatible with the database and thus their STs could not be defined. All *B. henselae* isolates with incompatible allelic profiles had the same allelic profile [*16 S rRNA* (allele 2), *batR* (allele 7), *groEL* (allele 1), *ftsZ* (allele 1), *gltA* (allele 1), *nlpD* (allele 1), *ribC* (allele 1), and *rpoB* (allele 1)] (Table 1). Depending on this result, we recommended a new sequence type called ST38. Chromatogram images belonging to these *B. henselae* isolates were given in Additional file 1:S2.

**Fig. 1:**
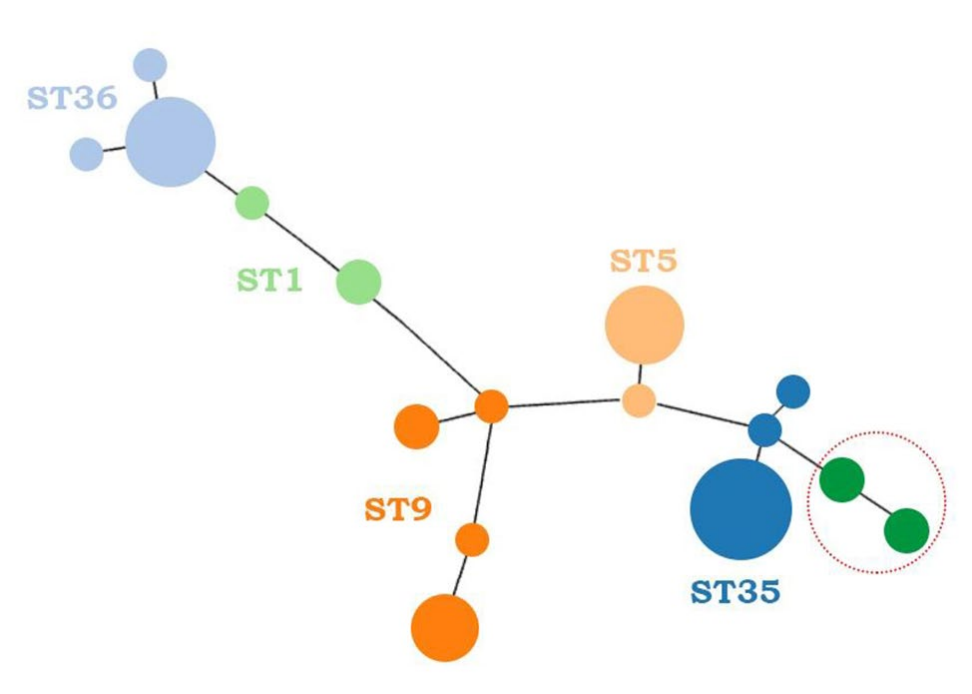
The PHYLOViZ results of *B. henselae* isolates. According to results of PHYLOViZ, many of ST35 isolates detected in this study except samples 482 and 380 were found to cluster with reference ST35 isolate detected in Spain. Among ST36 isolates, many of ST36 isolates detected in this study except samples 766 and 812 were found to cluster with reference ST36 isolate detected in Spain. Of the ST5 isolates detected in this study, many of them except sample 559 were found to cluster with ST5 isolate detected in New Zealand. ST9 isolates detected in this study were detected to have a higher variation and four of them were clustered with reference ST9 isolate detected in Germany whereas the remaining four ST9 isolates were closely clustered with reference ST9. One of ST1 isolates were clustered with reference ST1 detected in New Zealand whereas the other was closely clustered with reference ST1. *B. henselae* isolates (circled) with incompatible allelic profiles and called as ST38 were detected to cluster close to ST35 detected in Spain

**Fig. 2:**
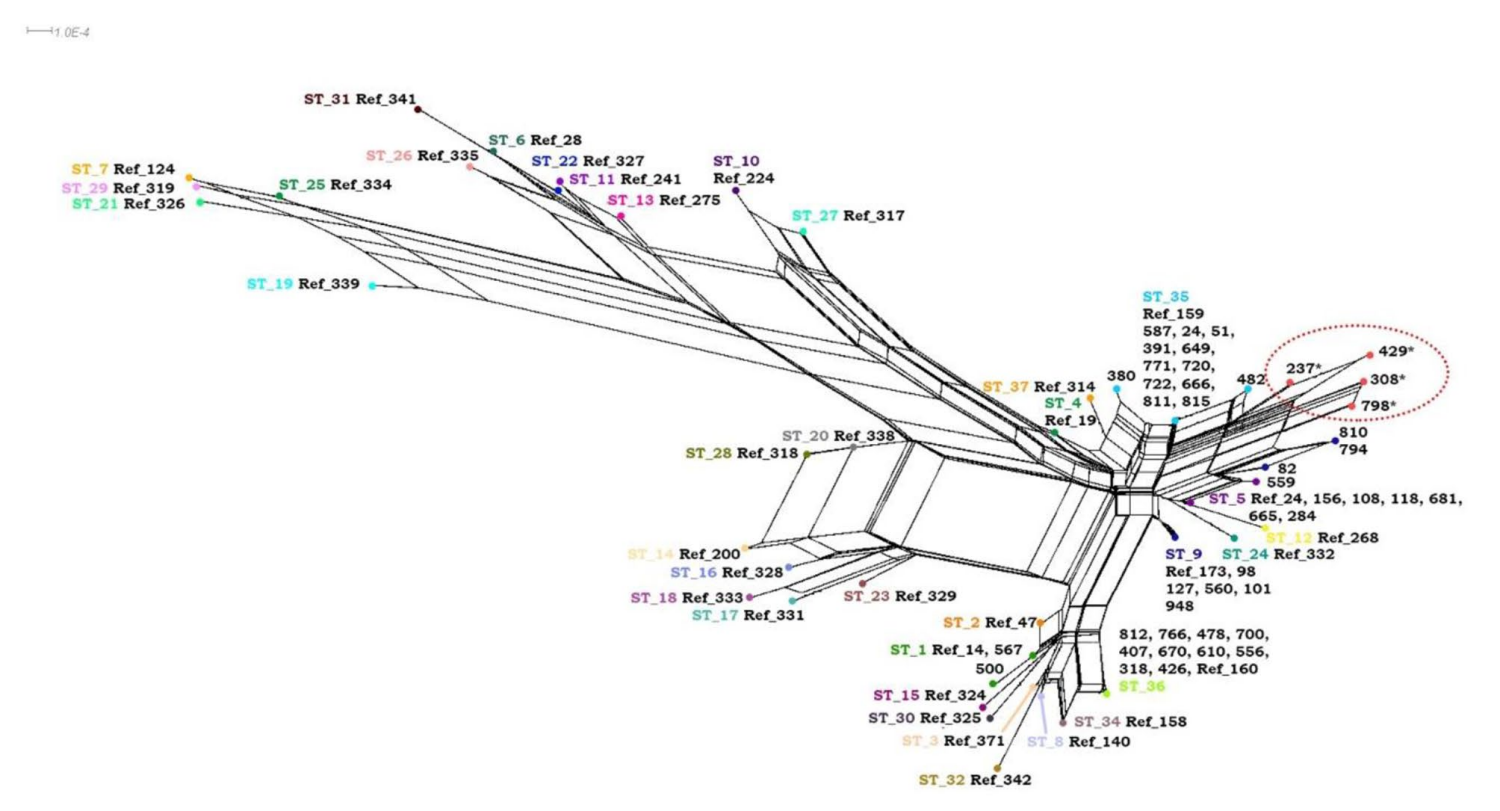
The SplitsTree results of *B. henselae* isolates. The SplitsTree showed that all of ST36 isolates detected in this study were clustered with reference ST36 isolate detected in Spain. Surprisingly, all of ST9 isolates were also detected to cluster with reference ST9 isolate detected in Germany contrary to PHYLOViZ result. However, results belonging to ST1, ST5, ST35 as well as *B. henselae* isolates with new allelic profile (circled) called as ST38 were same with results obtained from PHYLOViZ.

Concerning the *16 S rRNA*, 32 isolates (72.7%) were type II (Marseille) while the remaining 12 isolates (27.2%) were type I (type Houston I) (Table 1). Among *B. henselae* isolates with compatible allelic profiles, 5 different STs including ST1, ST5, ST9, ST35 and ST36 were detected by the *B. henselae* MLST online database. ST35 was the most prevalent ST with a prevalence rate of 29.5% (13/44), followed by ST36 with a prevalence rate of 22.7% (10/44). In addition, ST5 (16%, 7/44) and ST9 (18.2%, 8/44) were also among the prevalent STs. The prevalence of ST1 was detected as 4.5% (2/44). According to results obtained from PHYLOViZ online platform, many *B. henselae* isolates with compatible allelic profiles were detected to cluster with their own STs (Fig. 1). SplitsTree also confirmed that many *B. henselae* isolates clustered with their own STs (Fig. 2). In addition, *B. henselae* isolates with incompatible allelic profiles were detected to cluster closely to ST35 (Figs. 1 and 2).

**Table 1:** Allelic profiles of 44 *B. henselae* samples detected in stray cats according to the MLST database

Number of isolate	Sample Type	Host	*16 S*	*batR*	*ribC*	*nlpD*	*groEL*	*rpoB*	*gltA*	*ftzS*	ST
24	Blood	Feline	2	7	1	1	2	1	1	1	35
51	Blood	Feline	2	7	1	1	2	1	1	1	35
82	Blood	Feline	2	1	1	1	1	1	1	1	9
98	Blood	Feline	2	1	1	1	1	1	1	1	9
101	Blood	Feline	2	1	1	1	1	1	1	1	9
108	Blood	Feline	2	1	1	1	2	1	1	1	5
118	Blood	Feline	2	1	1	1	2	1	1	1	5
127	Blood	Feline	2	1	1	1	1	1	1	1	9
156	Blood	Feline	2	1	1	1	2	1	1	1	5
237	Blood	Feline	2	7	1	1	1	1	1	1	*
284	Blood	Feline	2	1	1	1	2	1	1	1	5
308	Blood	Feline	2	7	1	1	1	1	1	1	*
318	Blood	Feline	1	7	1	1	1	1	1	1	36
380	Blood	Feline	2	7	1	1	2	1	1	1	35
391	Blood	Feline	2	7	1	1	2	1	1	1	35
407	Blood	Feline	1	7	1	1	1	1	1	1	36
426	Blood	Feline	1	7	1	1	1	1	1	1	36
429	Blood	Feline	2	7	1	1	1	1	1	1	*
478	Blood	Feline	1	7	1	1	1	1	1	1	36
482	Blood	Feline	2	7	1	1	2	1	1	1	35
500	Blood	Feline	1	1	1	1	1	1	1	1	1
556	Blood	Feline	1	7	1	1	1	1	1	1	36
559	Blood	Feline	2	1	1	1	2	1	1	1	5
560	Blood	Feline	2	1	1	1	1	1	1	1	9
567	Blood	Feline	1	1	1	1	1	1	1	1	1
587	Blood	Feline	2	7	1	1	2	1	1	1	35
610	Blood	Feline	1	7	1	1	1	1	1	1	36
649	Blood	Feline	2	7	1	1	2	1	1	1	35
665	Blood	Feline	2	1	1	1	2	1	1	1	5
666	Blood	Feline	2	7	1	1	2	1	1	1	35
670	Blood	Feline	1	7	1	1	1	1	1	1	36
681	Blood	Feline	2	1	1	1	2	1	1	1	5
700	Blood	Feline	1	7	1	1	1	1	1	1	36
720	Blood	Feline	2	7	1	1	2	1	1	1	35
722	Blood	Feline	2	7	1	1	2	1	1	1	35
766	Blood	Feline	1	7	1	1	1	1	1	1	36
771	Blood	Feline	2	7	1	1	2	1	1	1	35
794	Blood	Feline	2	1	1	1	1	1	1	1	9
798	Blood	Feline	2	7	1	1	1	1	1	1	*
810	Blood	Feline	2	1	1	1	1	1	1	1	9
811	Blood	Feline	2	7	1	1	2	1	1	1	35
812	Blood	Feline	1	7	1	1	1	1	1	1	36
815	Blood	Feline	2	7	1	1	2	1	1	1	35
948	Blood	Feline	2	1	1	1	1	1	1	1	9

*indicates that *B. henselae* isolate has an incompatible allelic profile. These isolates have been called as ST38 in this study.

## Discussion

To date, in many studies conducted in different regions/countries, *B. henselae* isolates detected in humans or cats were genotyped to reveal their genetic diversity [1, 13–15]. Thanks to these studies, the number of STs within *B. henselae* isolates has increased from 7 to 37 [13, 14]. Although *B. henselae* isolates were detected in humans or cats in Turkey, they have not been genotyped before and thus, genotype profiles of these isolates are not known. In our previous study, the prevalence of *Bartonella* spp. in stray cats living in İzmir, Turkey were found to be 12.05% and among these positive *Bartonella* spp., *B. henselae*, *B. clarridgeiae* and *B. koehlerae* were identified [11]. In this study, previously identified *B. henselae* isolates were genotyped by MLST targeting *16 S rRNA*, *batR*, *ftsZ*, *gltA*, *groEL*, *nlpD*, *ribC*, and *rpoB* genes. According to the MLST results, ST1, ST5, ST9, ST35 and ST36 were detected among analyzed *B. henselae* isolates. The presence of ST1 and ST5 in stray cats was one of the important findings of this study in terms of their medical and veterinary importance because both of them have been associated with Cat Scratch Disease in humans and ST5 has been associated with feline infection [14]. The results of this study was comparable with previous studies and shows that ST1, ST5 and ST9 were frequently detected in cats [1, 12–18]. For example, of the 31 *B. henselae* samples isolated from domestic cats in Japan, 28 (90.3%) were identified as ST1 [17]. In another study, among 39 *B. henselae* samples isolated from cats in German, 10 (25.6%) were detected to be ST5 while 3 (7.69%) were detected to be ST1 [15]. In a study conducted in Spain, of the 21 *B. henselae* samples isolated from cats, 15 (71.4%) were identified as ST5 [12]. In a different study conducted in Argentina, of the 12 *B. henselae* samples isolated from cats, 7 (58.3%) were detected to be ST1 [18]. In Brazil, of the 12 *B. henselae* samples isolated from domestic cats, 11 (91.6%) were detected to be ST1 whereas 1 (8.3%) was found to be ST5 [1]. In a different study conducted in the same region, ST9 also was detected among *B. henselae* samples isolated from cats [14]. In a comprehensive study analyzing *B. henselae* samples isolated from cats in different regions such as Europe, USA and Australia, ST1 was detected with a prevalence rate of 17.1% while ST5 was detected with a prevalence rate of 20.9% [16]. In addition to these STs including ST1, ST5 and ST9, ST35 and ST36 were also reported to be detected in Spain [1].

Surprisingly, the allelic profile belonging to four *B. henselae* samples *16 S rRNA* (allele 2), *batR* (allele 7), *groEL* (allele 1), *ftsZ* (allele 1), *gltA* (allele 1), *nlpD* (allele 1), *ribC* (allele 1), and *rpoB* (allele 1)] (Table 1) was incompatible when compared with the available allelic profile present in MLST database and thus this strain was recommended as a new sequence type called ST38 in this study. Likewise, numerous new STs were detected in different regions in previous studies [12, 14–17, 19]. These previous results and our results support the idea that more studies are required to fully understand molecular epidemiology of *B. henselae* isolates.

Stray cats are animals whose population is on the rise due to owned cats either escaped or dumped on the street, and uncontrolled breeding of cats on the street. Some of these cats are imported and some come to the country by immigration from neighboring countries. The coexistence of these street-found or runaway cats facilitates the transmission of pathogens they carry to one another. Therefore, the import, migration, and mixing events observed in stray cats are thought to be potential mechanisms that can explain the ST diversity detected in *Bartonella* species and the presence of same predominant STs in diverse regions.

## Conclusion

This study genotyped for the first time *B. henselae* samples isolated from stray cats living in İzmir, Turkey using MLST method. ST1, ST5, ST9, ST35, ST36 as well as a new ST called ST38 were detected among *B. henselae* isolates. Depending on these results, it was thought that there is a wide *B. henselae* genetic diversity in Turkey and new studies analyzing more *B. henselae* isolates can be helpful to reveal new STs in stray cats in Turkey.

## Methods

### ***B. henselae*isolates**

A total of 44 *B. henselae* isolates previously detected in stray cats by sequencing ITS region [11] were used for genotyping by MLST analysis.

### MLST analysis

During MLST analysis, eight different housekeeping markers, including *16 S rRNA*, *batR*, *ftsZ*, *gltA*, *groEL*, *nlpD*, *ribC*, and *rpoB* loci were amplified by nested PCR and then sequenced for revealing STs of *B. henselae* isolates as previously described [13]. Briefly, each gene was amplified from *B. henselae* positive DNA samples using their specific primer pairs by a nested PCR. In the first reaction, the 25 µl reaction volume consisted of 1 µl template DNA, 1 µl of each primer (10 µM), 12.5 µl PCR master mix (GeneMark, Taichung, Taiwan) and 9.5 µl distilled water. In the second reaction, the same reaction condition was used except that 1 µl PCR product obtained from the first reaction was used as a template. During gene amplification, PCR was performed using the following calculated-control protocol: 5 min initial denaturation step at 96 ◦C, followed by 40 cycles of 10 s at 96 ◦C, 10 s at 55 ◦C, and 50 s at 72 ◦C, and a final extension of 10 min at 72 ◦C. After amplification, PCR products were visualized on 1% agarose gel, purified by the Qiaquick PCR Purification Kit (Qiagen, USA) and sequenced. Following this, the obtained sequences were analyzed by comparison with allelic profiles in the MLST database (https://pubmlst.org/organisms/Bartonella-henselae). The minimum spanning trees were created with the PHYLOViZ online platform (https://online.phyloviz.net/index#) using goeBURST algorithm along with reference examples from the database [20]. In addition, to determine the genetic distance between individuals, distance analysis (Split-Network) with Neighbor-net [21] method was created using the SplitsTree 4.11.3 program [22]. For the genetic analyses of *B. henselae* isolates, SplitsTree distance analysis was used previously by Furquim et al. [14], and the minimum spanning trees analysis by Dias et al. [1] and by Furquim et al. [14]. Reference samples containing a reference from each ST used during analyses were presented in Additional file 1:S1.

### Electronic supplementary material

Below is the link to the electronic supplementary material.

Supplementary Material 1

Supplementary Material 2

## Data Availability

All sequences obtained were deposited into GenBank (National Center for Biotechnology Information Search database). Provided GenBank accession numbers are as follows. OQ165187-OQ165188; OQ191232-OQ191240.

## Abbreviations

ST: Sequence type
MLST: Multi-locus sequence typing
ITS: Internal transcribed spacer gene
PCR: Polymerase chain reaction
