# Supplementary material for: Genetic characterization of Bartonella henselae samples isolated from stray cats by multi-locus sequence typing
Source: BMC Vet Res. 2023 Oct 7;19:195. doi: 10.1186/s12917-023-03748-4 (PMC10559530; doi:10.1186/s12917-023-03748-4)
Supplement: Supplementary file 1 — Supplementary Material 1 [file 12917_2023_3748_MOESM1_ESM.docx]

Table **S**1: Reference samples obtained from the PUBMLST database and used for SplitsTree distance analysis.

| PubMLST Sample ID | Host | ST | Country | Sample Code |
| --- | --- | --- | --- | --- |
| 14 | Feline | 1 | New Zealand | Ref_14 |
| 19 | Feline | 4 | Australia | Ref_19 |
| 24 | Feline | 5 | New Zealand | Ref_24 |
| 28 | Feline | 6 | Australia | Ref_28 |
| 47 | Feline | 2 | UK | Ref_47 |
| 124 | Feline | 7 | UK | Ref_124 |
| 140 | Feline | 8 | UK | Ref_140 |
| 158 | Human | 34 | Spain | Ref_158 |
| 159 | Human | 35 | Spain | Ref_159 |
| 160 | Human | 36 | Spain | Ref_160 |
| 173 | Feline | 9 | Germany | Ref_173 |
| 200 | Feline | 14 | Germany | Ref_200 |
| 224 | Feline | 10 | UK | Ref_224 |
| 241 | Feline | 11 | France | Ref_241 |
| 268 | Feline | 12 | Israel | Ref_268 |
| 275 | Feline | 13 | Czech Republic | Ref_275 |
| 317 | Feline | 27 | UK | Ref_317 |
| 318 | Feline | 28 | UK | Ref_318 |
| 319 | Feline | 29 | UK | Ref_319 |
| 324 | Feline | 15 | Japan | Ref_324 |
| 325 | Feline | 30 | China | Ref_325 |
| 326 | Feline | 21 | Germany | Ref_326 |
| 328 | Feline | 16 | Germany | Ref_328 |
| 331 | Feline | 17 | Germany | Ref_331 |
| 333 | Feline | 18 | Germany | Ref_333 |
| 327 | Feline | 22 | Germany | Ref_327 |
| 329 | Feline | 23 | Germany | Ref_329 |
| 332 | Feline | 24 | Germany | Ref_332 |
| 334 | Feline | 25 | Germany | Ref_334 |
| 335 | Feline | 26 | Germany | Ref_335 |
| 338 | Feline | 20 | Germany | Ref_338 |
| 339 | Feline | 19 | Germany | Ref_339 |
| 341 | Feline | 31 | Spain | Ref_341 |
| 342 | Feline | 32 | Spain | Ref_342 |
| 371 | Feline | 3 | Spain | Ref_371 |
| 447 | Feline | 33 | Croatia | Ref_447 |

Table **S**2: Reference samples obtained from the PubMLST and used for Phyloviz analysis.

| Sample ID: | Host | ST | Country: | Sample Code |
| --- | --- | --- | --- | --- |
| 14 | Feline | 1 | New Zealand | Ref_14 |
| 24 | Feline | 5 | New Zealand | Ref_24 |
| 159 | Human | 35 | Spain | Ref_159 |
| 160 | Human | 36 | Spain | Ref_160 |
| 173 | Feline | 9 | Germany | Ref_173 |
