# Supplementary material for: Genetic characterization of Bartonella henselae samples isolated from stray cats by multi-locus sequence typing
Source: BMC Vet Res. 2023 Oct 7;19:195. doi: 10.1186/s12917-023-03748-4 (PMC10559530; doi:10.1186/s12917-023-03748-4)
Supplement: Supplementary file 2 — Supplementary Material 2 [file 12917_2023_3748_MOESM2_ESM.pdf]

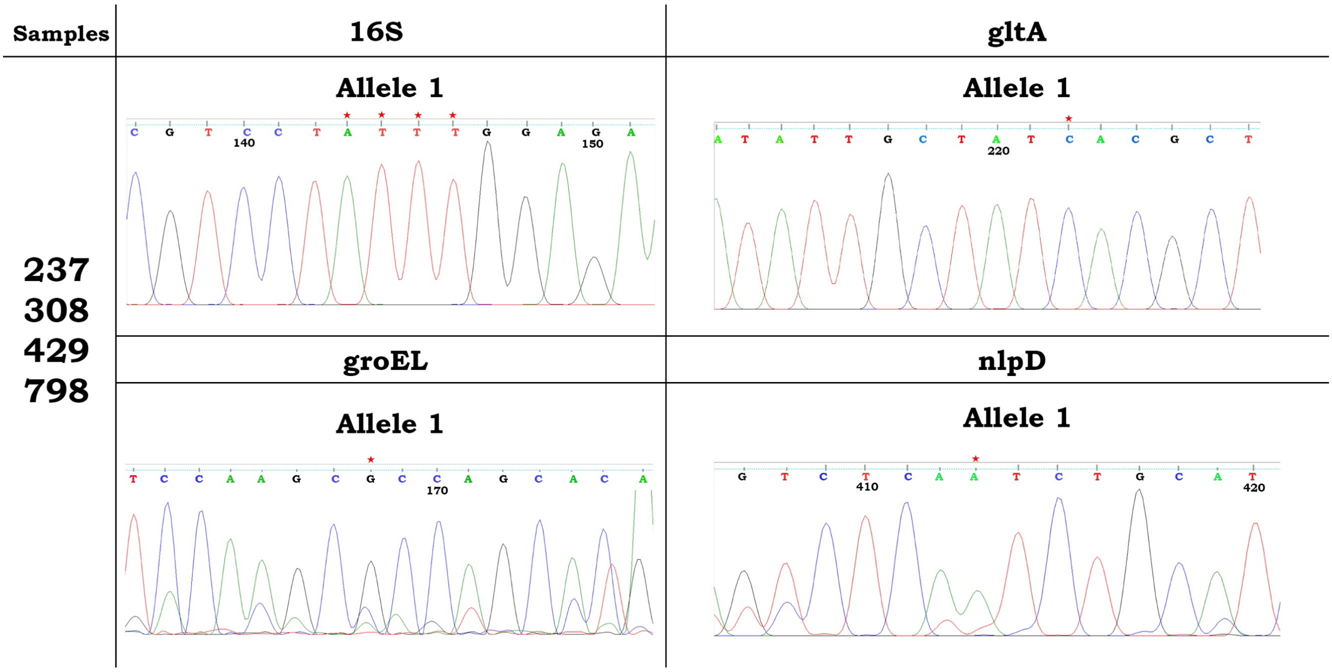

Figure S1. Sequencing chromatograms belonging to *16S*, *gltA*, *groEL* and *nlpD*.
*shows the position used for identification of allele.

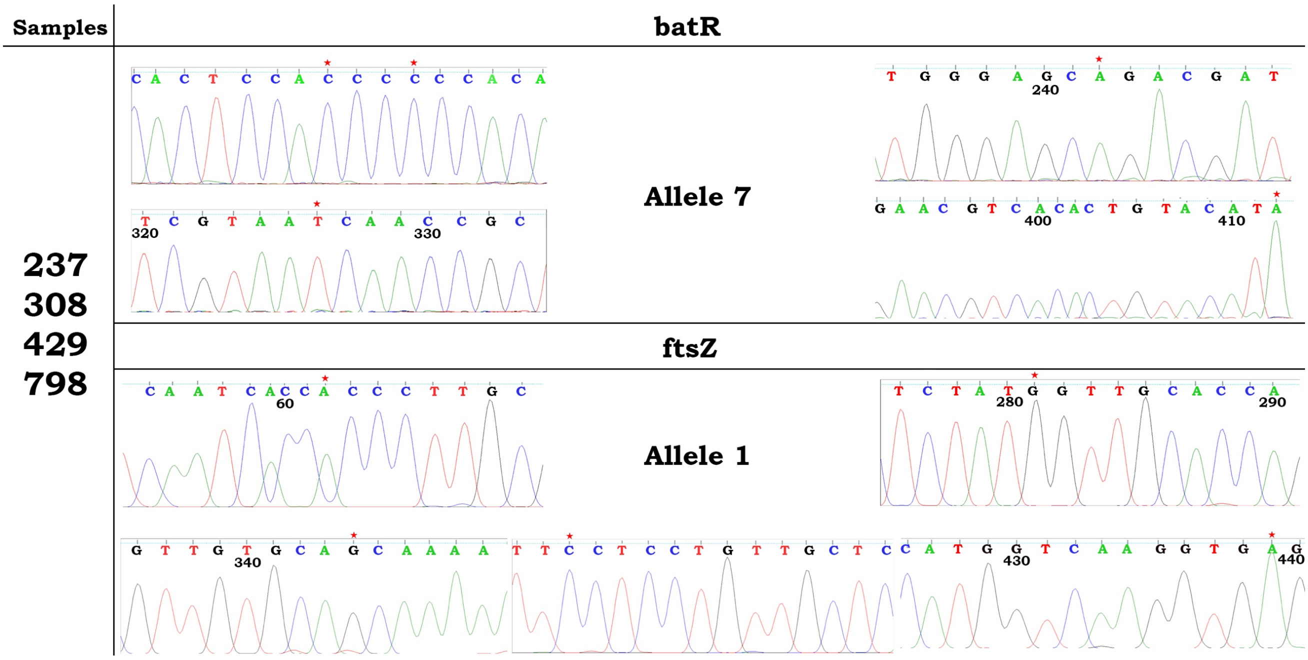

Figure S2. Sequencing chromatograms belonging to *batR* and *ftsZ*.
*shows the position used for identification of allele.

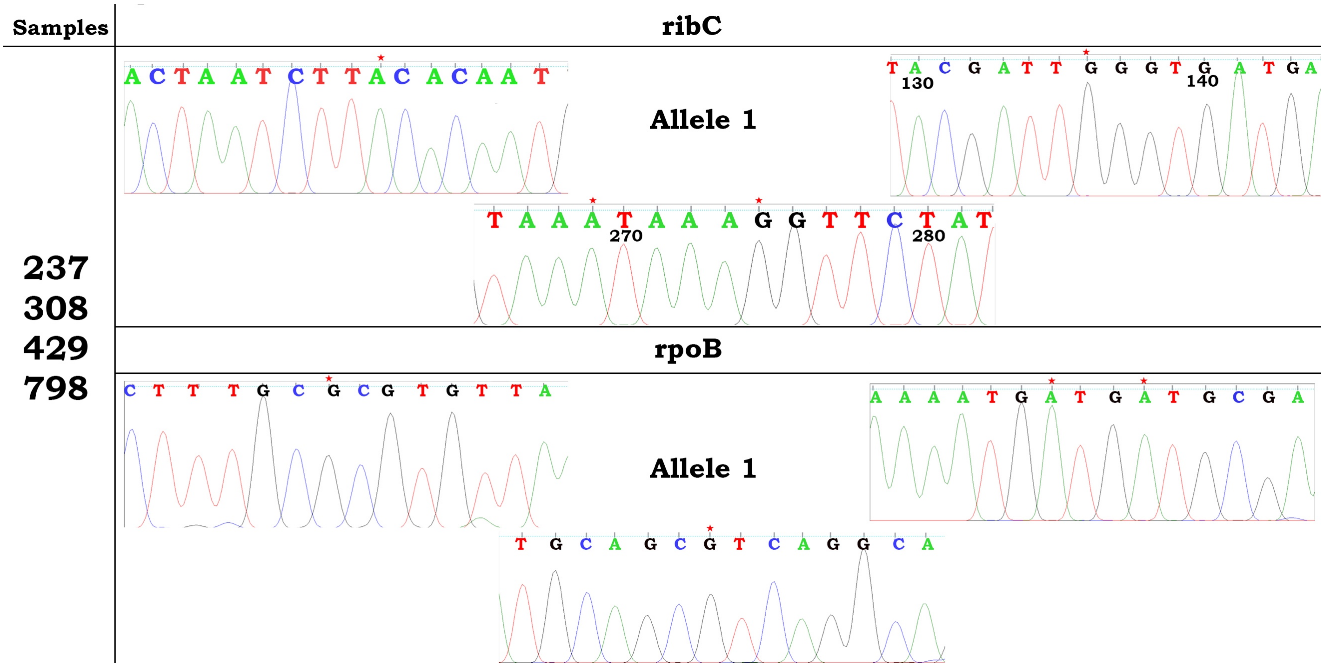

Figure **S**3. Sequencing chromatograms belonging to *ribC* and *rpoB*.
*shows the position used for identification of allele.
